# Patterns of lymph node metastasis in level IIB and contralateral level VI for papillary thyroid carcinoma with pN1b and safety of low collar extended incision for neck dissection in level II

**DOI:** 10.1186/s12957-023-03075-w

**Published:** 2023-08-17

**Authors:** Yudong Ning, Yuebai Liu, Dingfen Zeng, Yuqiu Zhou, Linjie Ma, Shuang Dong, Jianfeng Sheng, Gaosong Wu, Wen Tian, Yongcong Cai, Chao Li

**Affiliations:** 1 Department of Head and Neck Surgery, Sichuan Cancer Hospital & Institute, Sichuan Cancer Center, University of Electronic Science and Technology of China, , Chengdu, China; 2 Department of Head and Neck Surgery, Education & Training, Sichuan Cancer Center, University of Electronic Science and Technology of China, Chengdu, China; 3 Department of Thyroid, Head, Neck and Maxillofacial Surgery, The Third People’s Hospital of Mianyang, Sichuan Mental Health Center, Mianyang, China; 4 Department of Thyroid and Breast Surgery, Zhongnan Hospital of Wuhan University, Wuhan, China; 5 Department of General Surgery, Chinese PLA General Hospital, Beijing, China

**Keywords:** Papillary thyroid carcinoma, Lateral cervical lymphatic metastasis, Contralateral central lymphatic metastasis, Low-collar extended incision

## Abstract

**Objective:**

To explore relevant clinical factors of level IIB and contralateral level VI lymph node metastasis and evaluate the safety of low-collar extended incision (LCEI) for lymph node dissection in level II for papillary thyroid carcinoma (PTC) with pN1b.

**Method:**

A retrospective analysis was performed on 218 patients with PTC with pN1b who were treated surgically in the Head and Neck Surgery Center of Sichuan Cancer Hospital from September 2021 to May 2022. Data on age, sex, body mass index (BMI), tumor location, maximum tumor diameter, multifocality, Braf gene, T staging, surgical incision style, and lymph node metastasis in each cervical subregion were collected. The chi-square test was used for comparative analysis of relevant factors. All statistical analyses were completed by SPSS 24 software.

**Result:**

Each subgroup on sex, age, BMI, multifocality, tumor location, extrathyroidal extension, Braf gene, and lymphatic metastasis in level III, level IV, and level V had no significant difference in the positive rate of lymph node metastasis in level IIB (*P* > 0.05). In contrast, patients with bilateral lateral cervical lymphatic metastasis were more likely to have level IIB lymphatic metastasis than those with unilateral lateral cervical lymphatic metastasis, with a statistically significant difference (*P* = 0.000). In addition, lymph node metastasis in level IIA was significantly associated with lymph node metastasis in level IIB (*P* = 0.001). After multivariate analysis, lymph node metastasis in level IIA was independently associated with lymph node metastasis in level IIB (*P* = 0.010). The LCEI group had a similar lymphatic metastasis number and lymphatic metastasis rate in both level IIA and level IIB as the L-shaped incision group (*P* > 0.05). There were 86 patients with ipsilateral central lymphatic metastasis (78.2%). Patients with contralateral central lymphatic metastasis accounted for 56.4%. The contralateral central lymphatic metastasis rate was not correlated with age, BMI, multifocality, tumor invasion, or ipsilateral central lymphatic metastasis, and there was no significant difference (*P* > 0.05). The contralateral central lymphatic metastasis in males was slightly higher than that in females, and the difference was statistically significant (68.2% vs. 48.5%, *P* = 0.041).

**Conclusion:**

Lymphatic metastasis in level IIA was an independent predictor of lymphatic metastasis in level IIB. When bilateral lateral cervical lymphatic metastasis or lymph node metastasis of level IIA is found, lymph node dissection in level IIB is strongly recommended. When unilateral lateral cervical lymphatic metastasis and lymphatic metastasis in level IIA are negative, lymph node dissection in level IIB may be performed as appropriate on the premise of no damage to the accessory nerve. LCEI is safe and effective for lymph node dissection in level II. When the tumor is located in the unilateral lobe, attention should be given to contralateral central lymph node dissection because of the high lymphatic metastasis rate.

## Introduction

PTC is a common endocrine tumor [1]. Cervical lymph node metastasis is very common in PTC and is an important factor in predicting the recurrence and prognosis of the tumor [2].The American Thyroid Association recommended therapeutic lateral neck dissection for PTC with lateral cervical lymphatic metastasis [3]. Considering both functional and radical resection of the tumor, most scholars support functional neck dissection that preserves the sternocleidomastoid muscle, internal jugular vein, and accessory nerve (level II–level V) [4, 5]. However, the extent of lymph node dissection is not clear. Shoulder dysfunction caused by accessory nerve injury is one of the main complications of lateral neck dissection [6]. The dissection of the lateral cervical lymph node related to the accessory nerve is mainly due to lymph node dissection at level IIB because the accessory nerve is the dividing line between level IIA and level IIB, and the anatomical position of level IIB is on the upper side, resulting in a narrow anatomical space and difficult exposure. In that situation, the accessory nerve is susceptible to strain and damage [7]. Moreover, a few studies have reported a low rate of lymphatic metastasis in level IIB, and the controversial point of the extent of neck dissection is whether lymph node dissection in level IIb is performed routinely [8].

At present, the two main incision styles for lateral cervical lymph node dissection of PTC include LCEI and L-shaped incision [9, 10]. An L-shaped incision can fully expose the lymph nodes in level II so that complete dissection of the lymph nodes can be achieved. However, this incision can cause scarring because part of the incision is perpendicular to the neck dermatoglyph [11]. In contrast, the LCEI is in the direction of the dermatoglyph, which can reduce surgical scars and improve the demand of patients for cosmetic surgery. However, as the incision location is far from level II, it is controversial whether it can fully expose the lymph nodes in level II and perform complete lymph node dissection [12].

PTC is prone to central lymph node metastasis. Central lymph nodes can be divided into ipsilateral central lymph nodes and contralateral central lymph nodes [13]. Routine prophylactic central lymph node dissection is required [3]. However, for unilateral cancer, dissection of the contralateral central lymph node is controversial, mainly because of the low metastasis rate and postoperative complications [14, 15]. For PTC with N1b, there are few studies on contralateral central lymph node dissection of unilateral cancer.

In this study, PTC with pN1b was studied to answer the above three questions to make better clinical decisions.

## Method

### Characteristics of the cohort

A retrospective analysis was performed on 218 patients with PTC with pN1b who were treated by surgery in the Head and Neck Surgery Center of Sichuan Cancer Hospital from September 2021 to May 2022. The data on age, sex, BMI, tumor location, maximum tumor diameter, multifocality, Braf gene, T staging, surgical incision style, and lymphatic metastasis in each cervical subregion were collected. T staging was performed according to the eighth edition of staging [16]. There are unilateral and bilateral cancers based on tumor location. Multifocal carcinoma was defined as having two or more nodes in at least one lobule.

### Diagnostic evaluation

Color ultrasound examination was performed before surgery. Patients with suspected lymph node metastasis were confirmed to have lateral cervical lymph node metastasis in papillary thyroid carcinoma by fine needle puncture or intraoperative freezing. The final diagnosis is based on postoperative pathology.

### Surgical method

**Fig. 1:**
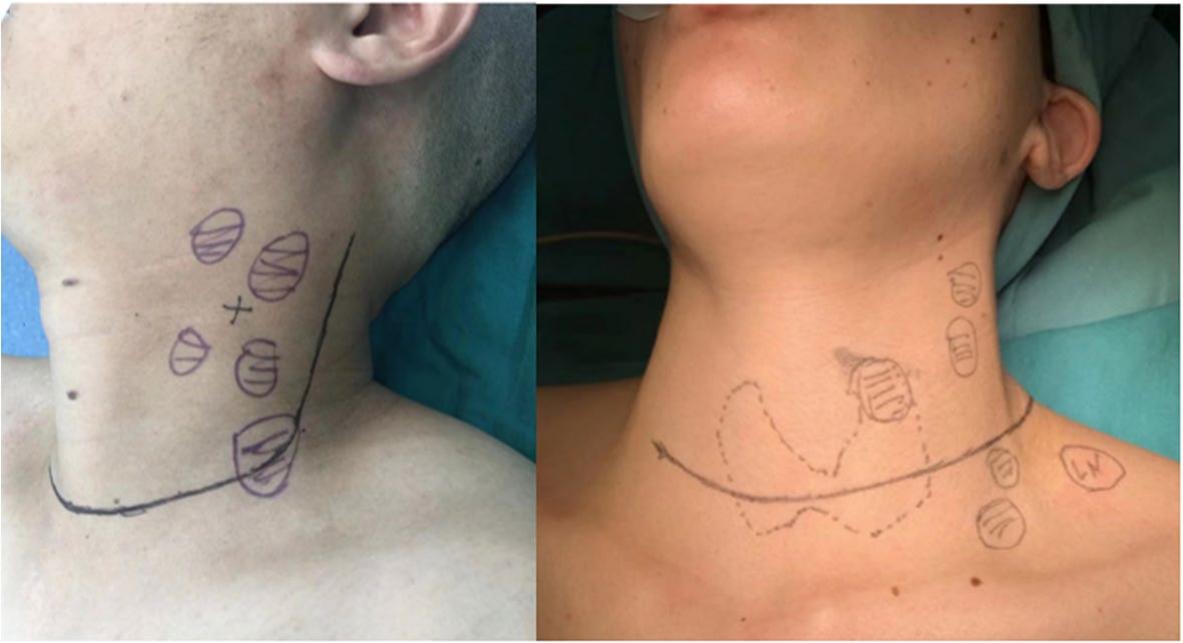
**A** (right) An L-shaped incision is a transverse arc-shaped incision along the dermatoglyph at the distance of one to two transverse fingers above the supraclavicular notch, extending to the posterior margin of sternocleidomastoid, then up to the mastoid process. **B** (right) The low-collar extended incision is a transverse arc-shaped incision along the dermatoglyph at the distance of one to two transverse fingers above the supraclavicular notch, extending to the anterior edge of the trapezius muscle

**Fig. 2:**
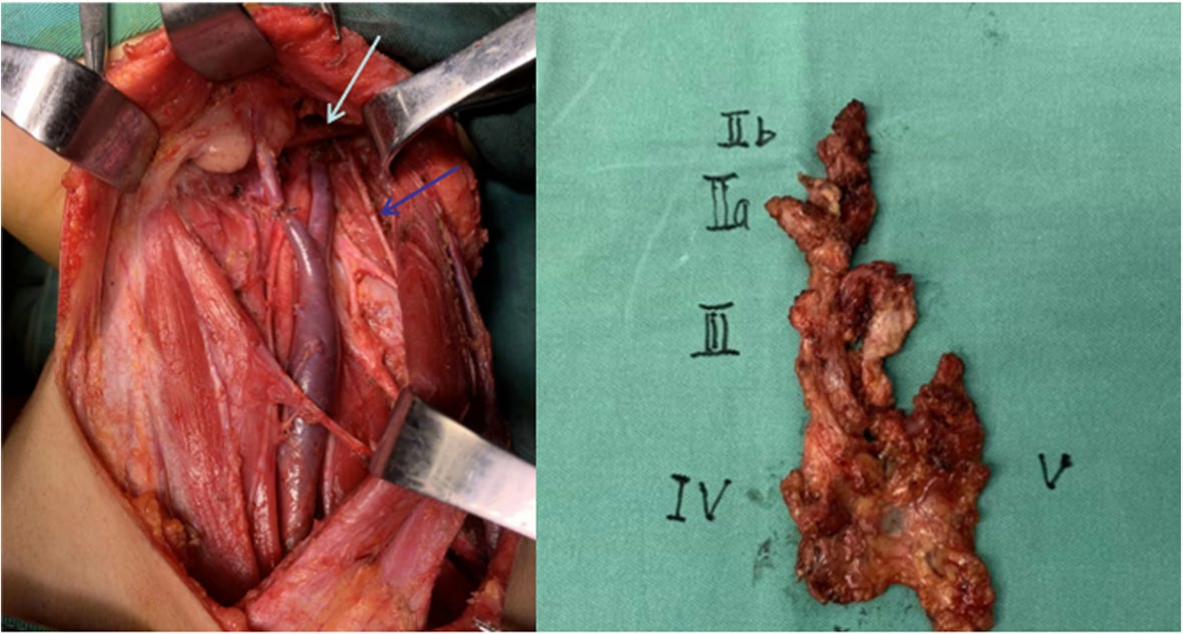
**A** Anatomical display after dissection with low-collar extended incision. The white arrow points to the posterior belly of the digastric muscle. The blue arrow points to the accessory nerve. **B** (right) The lateral cervical lymph nodes were divided into IIA, IIB, III, IV, and V

All patients underwent total thyroidectomy plus central and lateral neck dissection. The lymph nodes in the central region were divided into left tracheal esophageal sulcus, right tracheal esophageal sulcus, anterior laryngeal and anterior tracheal lymph nodes, and the lateral cervical lymph nodes were divided into IIA, IIB, III, IV, and V (Fig. 1B). The incision styles are divided into LCEI (Fig. 2B) and L-shaped incisions (Fig. 2A). The LCEI is a transverse arc-shaped incision along the dermatoglyph one to two transverse fingers above the supraclavicular notch, extending to the anterior edge of the trapezius muscle. An L-shaped incision is a transverse arc-shaped incision along the dermatoglyph at a distance of one to two transverse fingers above the supraclavicular notch, extending to the posterior margin of the sternocleidomastoid and then up to the mastoid process. The choices of the incision styles depend on the surgeon's personal preferences.

### Statistical method

We used univariate analysis to analyze the association between characteristics of the cohort and lymphatic metastasis in level IIB and then found the related risk factors, which were further analyzed by multivariate analysis (Table 1). We analyzed the association between characteristics of the cohort and incision styles and compared the positive lymph node numbers and ratios in levels IIA and IIB to the safety of low-collar extended incision for lymph node dissection in level II. Additionally, we analyzed the association between characteristics of the cohort and the positive number of contralateral central cervical lymph nodes for unilateral cancer. The chi-square test was used for all comparative analyses of relevant factors. All statistical analyses were completed by SPSS 24 software.

**Table 1:** The association between characteristics of the cohort and lymphatic metastasis in level IIB

Clinical characters	Number(%)	Lymph node positive number in level IIB(%)	Univariate *P* value	Multivariate *P* value
Gender				
Female	141(64.7)	15(10.6)	0.289	–
Male	77(35.3)	12(15.6)		
Age				
< 55	198(90.8)	22(11.1)	0.072	–
> = 55	20(9.2)	5(25.0)		
BMI				
< = 23.9	129(59.2)	16(12.4)	0.992	–
> 23.9	89(40.8)	11(12.3)		
Tumor sides				
Unilateral cancer	137(62.8)	15(10.9)	0.402	–
Bilateral cancer	81(37.2)	12(14.8)		
Multifocality				
No	152(69.7)	19(12.5)	0.938	–
Yes	66(30.3)	8(12.1)		
T stage			0.549	
T1,2,3a	207(95)	25(12.1)		–
T3b,4a,4b	11(5)	2(18.2)		
Braf(20 cases)			0.274	
Negative	7(35.0)	0(0)		–
Positive	13(65.0)	2(15.4)		
PN1b				
Unilateral lymph node metastasis	187(85.8)	15(8.0)	0.000	0.159
Bilateral lymph node metastasis	31(14.2)	12(38.7)		
IIA Positive			0.001	
No	111(50.9)	6(5.4)		0.010
Yes	107(49.1)	21(19.6)		
IIB Positive				
No	191(87.6)			
Yes	27(12.4)			
III Positive			0.808	
No	44(20.2)	5(11.4)		
Yes	173(79.4)	22(12.7)		–
IV Positive			0.476	
No	61(28)	6(9.8)		–
Yes	157(72)	21		
V Positive			0.186	
No	211(96.8)	25(11.8)		–
Yes	7(3.2)	2(28.6)		

## Result

### The characteristics of the cohort

**Fig. 3:**
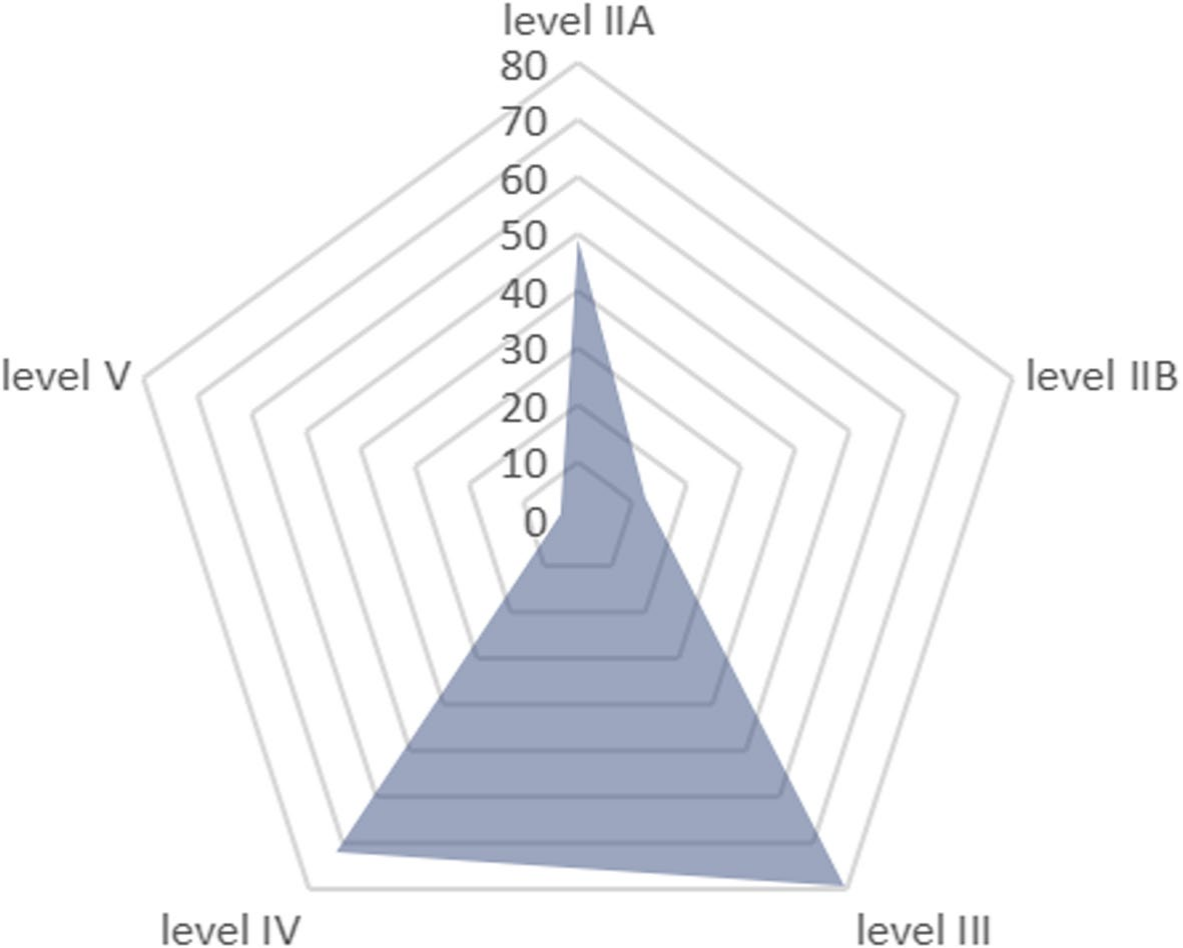
Radar map:the percentage of lymph node metastasis in different subregions of the lateral neck

A total of 218 patients were included in the study (Table 1). There were 141 cases (64.7%) in males and 77 cases (35.3) in females. The age cohort was divided into two groups: < 55 years old group and >  = 55 years old group. The former group had 198 cases (90.8%), and the latter group had 20 cases (9.2%). Based on BMI, all patients were divided into a nonobese group (BMI <  = 23.9) and an obese group (BMI > 23.9), accounting for 129 (59.2%) and 89 (40.8%) patients, respectively. The number of unilateral and bilateral cancers was 137 (62.8%) and 81 (37.2%), respectively. The number of cases with a single cancer was 152 (69.7%), and the number of cases with multiple cancers was 66 (30.3%). According to the primary tumor stage, all patients were divided into two groups: intrathyroidal extension group (T1, 2, 3a) and gross extrathyroidal extension group (T3b, 4A, 4b), with 207 cases (95%) in the former and 11 cases (5%) in the latter. The Braf gene was detected in 20 patients; 7 (35.0%) were negative, and 13 (65.0%) were positive. In all patients with pN1b, there were 187 cases (85.8%) with unilateral lateral cervical lymphatic metastasis and 31 cases (14.2%) with bilateral lateral cervical lymphatic metastasis. There were 107 cases (49.1%) of lateral cervical lymphatic metastasis in level IIA, 27 cases (12.4%) of lymphatic metastasis in level IIB, 174 (79.4%) of lymphatic metastasis in level III, and 157 (72%) of lymphatic metastasis in level IV. In addition, there were 7 patients (3.2%) with positive cervical lymph nodes in level V (Fig. 3).

### The association between characteristics of the cohort and lymphatic metastasis in level IIB

Meanwhile, we compared and analyzed the differences in the positive rate of lymph nodes in level IIB and above subgroups. The results showed that in each subgroup on gender, age, BMI, multifocality, tumor location, extrathyroidal extension, Braf gene, level III, level IV, and level V, lymphatic metastasis had no significant difference in the positive rate of lymph node metastasis in level IIB (*P* > 0.05). In contrast, patients with bilateral lateral cervical lymphatic metastasis were more likely to have lymphatic metastasis in level IIB than those with unilateral lateral cervical lymphatic metastasis, with a statistically significant difference (*P* = 0.000). In addition, level IIA lymphatic metastasis was significantly associated with level IIB lymphatic metastasis (*P* = 0.001). After multivariate analysis, lymph node metastasis in level IIA was independently associated with lymph node metastasis in level IIB (*P* = 0.010) (Table 1).

### Safety of low-collar extended incision for lymph node dissection in level II dissection

In terms of surgical incision design, one case was endoscopic-assisted dissection of the lateral lymph node, which was excluded. Of the remaining 217 patients, 81 (37.3%) underwent an L-shaped incision, and 136 (62.7%) underwent LCEI. There were no significant differences between the two incision styles in sex, age, BMI, tumor location, multifocality or tumor invasion (*P* > 0.05). More importantly, there were no significant differences in lymph node metastasis number and lymph node metastasis rate between the two groups in either level IIA or level IIB (*P* > 0.05) (Table 2).

**Table 2:** The association between characteristics of the cohort and incision style

Clinical characters	L- shaped incision Number(%)	Low-collar incision number number(%)	*P* value
Gender			
Female	54(66.7)	86(63.2)	0.609
Male	27(33.3)	50(36.8)	
Age			
< 55	73(90.1)	124(91.2)	0.795
> = 55	8(9.9)	12(8.8)	
BMI			
< = 23.9	48(59.3)	80(58.8)	0.950
> 23.9	33(40.7)	56(41.2)	
Tumor sides			
Unilateral side	54(66.7)	82(58.8)	0.348
Bilateral side	27(33.3)	54(41.2)	
Mulifocality			
No	55(67.9)	96(70.6)	0.677
Yes	26(32.1)	40(29.4)	
T stage			0.858
T1,2,3a	77(95.1)	130(95.6)	
T3b,4a,4b	4(4.9)	6(4.4)	
IIA Positive			0.0792
No	42(51.9)	68(50.0)	
Yes	39(48.1)	68(50.0)	
IIB Positive			0.576
No	70(86.4)	121(89.0)	
Yes	11(13.6)	15(11.0)	
IIA positive lymph node ratio(average)	0.152	0.106	0.062
IIB positive lymph node ratio(average)	0.612	0.321	0.154

### The association between characteristics of the cohort and the positive lymph node number of contralateral central lymph nodes for unilateral cancer

We screened all patients whose tumors were in unilateral lobes and underwent bilateral central lymph node dissection. There were a total of 110 patients (Table 3). There were 66 cases (60.0%) in males and 44 cases (40.0%) in females. The age cohort was divided into two groups: < 55 years old group and >  = 55 years old group. The former group had 103 cases (93.6%), and the latter group had 7 cases (6.4%). Based on BMI, all patients were divided into a nonobese group (BMI <  = 23.9) and an obese group (BMI > 23.9), accounting for 63 (57.3%) and 47 (42.7%) patients, respectively. The number of patients with a single cancer was 85 (77.3%), and the number of patients with multiple cancers was 25 (22.7%). According to the primary tumor stage, all patients were divided into two groups: intrathyroidal extension (T1, 2, 3a) and gross extrathyroidal extension (T3b, 4A, 4b), with 106 cases (96.4%) in the former and 4 cases (3.6%) in the latter. There were 86 patients with ipsilateral, central lymphatic metastasis, with a percentage of 78.2%. Patients with contralateral central lymphatic metastasis accounted for 56.4%, with a total of 62. In addition, the contralateral central lymphatic metastasis rate was not correlated with age, BMI, multifocality, tumor invasion, or ipsilateral central lymphatic metastasis, and there was no significant difference (*P* > 0.05). The contralateral central lymphatic metastasis in males was slightly higher than that in females, and the difference was statistically significant (68.2% vs. 48.5%, *P* = 0.041) (Table 3).

**Table 3:** The association between characteristics of the cohort and the positive number of the contralateral central cervical lymph node for unilateral cancer

Clinical characters	Number(%)	Contralateral central lymph node positive Number(%)	*P* value
Gender			
Female	66(60.0)	32(48.5)	0.041
Male	44(40.0)	30(68.2)	
Age			
< 55	103(93.6)	58(56.3)	0.966
> = 55	7(6.4)	4(57.1)	
BMI			
< = 23.9	63(57.3)	33(52.4)	0.329
> 23.9	47(42.7)	29(61.7)	
Multifocality			
No	85(77.3)	48(56.5)	0.967
Yes	25(22.7)	14(56.0)	
T stage			0.794
T1,2,3a	106(96.4)	60(56.6)	
T3b,4a,4b	4(3.6)	2(50.0)	
Ipsilateral central cervical lymphatic metastasis			
No	24(21.8)	11(45.8)	0.239
Yes	86(78.2)	51(59.3)	
Contralateral central cervical lymph node			
No	48(43.6)		
Yes	62(56.4)		

## Discussion

In the current literature, the metastatic rate of IIB lymph nodes in PTC is 2.1–22.0% [17–20]. Our rate is 12.4%. Lymph node dissection in level IIB is likely to lead to shoulder disorders with an incidence of 1.5–27% [5, 21]. Therefore, whether lymph node dissection in level IIB is routinely performed is controversial. Some scholars have analyzed the risk factors for lymphatic metastasis in level IIB to identify some predictors. Lee et al. reported that if lymph nodes of IIA were negative, the incidence of metastasis of level IIB was low [20]. Two other studies reported that extensive involvement of the lateral cervical lymph node is a risk factor for predicting lymphatic metastasis in level IIB [19, 22]. In our study, patients with bilateral lateral lymphatic metastasis were more likely to have level IIB lymphatic metastasis than those with unilateral cervical lymphatic metastasis, with a statistically significant difference (*P* = 0.000). In addition, lymphatic metastasis in level IIA was significantly associated with lymphatic metastasis in level IIB (*P* = 0.001) (Table 1). However, 111 patients were negative for lymph nodes in level IIA, among which 6 patients (5.4%) were positive for level IIB with a small rate. Therefore, when bilateral lateral cervical lymphatic metastasis or lymph node metastasis is level IIA, lymph node dissection in level IIB is strongly recommended. When unilateral lateral cervical lymphatic metastasis and lymphatic metastasis in level IIA are negative, lymph node dissection in level IIB may be performed as appropriate on the premise of no damage to the accessory nerve.

For level IIB, we also need to consider the impact of the surgical incision style because it may determine whether lymph nodes in level IIB are fully exposed and dissected. To improve the satisfaction of the patient’s appearance, a variety of surgical incisions have been designed for PTC [23–25]. There are two common incision styles for lateral cervical lymph node dissection: the traditional L-shaped incision (Fig. 2A) and LCEI (Fig. 2B). The traditional L-shaped incision has a large scope of flaps. After the sternocleidomastoid muscle is completely dissociated and lifted, all neck areas are well exposed, which is convenient for anatomical preservation of cranial nerves and neck great vessels and other important structures. It is a surgical incision that is relatively mature and easy to master. However, the longitudinal part of the incision was perpendicular to the dermatoglyph, the incision suture had large tension and poor appearance in the healing process, and longitudinal scar contracture could cause varying degrees of restricted activity of the neck. LCEI is parallel to the dermatoglyph, avoiding the longitudinal incision. Although the difficulty and time of the surgery are increased, the postoperative appearance is good, the discomfort is light, and it has obvious advantages over the traditional L-shaped incision. However, the controversial point lies in the complete exposure of lymph nodes in level II. In our study, we specifically analyzed the comparison of the dissection of lymph nodes in level II by two incision styles. A total of 217 patients were included in our study: 81 (37.3%) underwent an L-shaped incision, and 136 (62.7%) underwent LCEI. There were no significant differences between the two incision styles in sex, age, BMI, tumor location, multifocality or tumor invasion (*P* > 0.05). More importantly, there were no significant differences in lymphatic metastasis number and lymphatic metastasis rate between the two groups in either level IIA or level IIB (*P* > 0.05). Moreover, the anatomical area of level II can also be fully exposed through LCEI (Fig. 1A). Thus, LCEI is a safe and effective incision that takes into account both radical tumor treatment and patient function. In other words, different surgical incisions selected by surgeons with different interests do not result in different lymph node dissections in level IIB.

Contralateral central lymph node dissection for unilateral PTC is controversial, mainly because of its low metastasis rate and the occurrence of complications such as hypoparathyroidism and recurrent laryngeal nerve injury. Therefore, some scholars have looked for predictors of contralateral central cervical lymphatic metastasis [14, 26]. Han et al. created a prediction model for contralateral central cervical lymphatic metastases in unilateral PTC [27]. Kim et al. suggested that ipsilateral central lymphatic metastasis could predict contralateral central lymphatic metastasis. In addition, they demonstrated a strong association between contralateral central lymphatic metastasis and specific clinicopathological features, including young male sex, multifocality, external thyroid extension, and tumor size [28]. However, for patients with PTC with CN1b, total thyroidectomy plus lateral cervical lymph node dissection is generally performed. Few studies have reported whether contralateral central lymph node dissection is necessary for patients with unilateral PTC with N1b. In our study, the number of patients with ipsilateral central lymphatic metastasis was 78.2%. Patients with contralateral central lymphatic metastasis accounted for 56.4%. In addition, the contralateral central lymphatic metastasis rate was not correlated with age, BMI, multifocality, tumor invasion or ipsilateral central lymphatic metastasis, and there was no significant difference (*P* > 0.05). Interestingly, the contralateral central lymphatic metastasis in males was slightly higher than that in females, and the difference was statistically significant (68.2% vs. 48.5%, *P* = 0.041) (Table 3). Thus, for patients with PTC with pN1b, contralateral central lymph node dissection is recommended, considering the high lymphatic metastasis rate.

This study has two limitations. First, in this study, the lymphatic metastasis rate of unilateral cervical and bilateral cervical level IIB was not carefully and clearly calculated and divided, nor were the effects of ipsilateral cancer on ipsilateral and contralateral cervical node metastasis. Second, the overall sample size is small, and there may be some bias. Third, we only assessed the integrity of LCEI for lymph node dissection but did not assess the satisfaction of patients or accessory nerve functional status.

## Conclusion

Lymphatic metastasis in level IIA was an independent predictor of lymphatic metastasis in level IIB. When there is bilateral lateral cervical lymphatic metastasis or lymph node metastasis of level IIA, lymph node dissection in level IIB is strongly recommended. When unilateral lateral cervical lymphatic metastasis and lymphatic metastasis in level IIA are negative, lymph node dissection in level IIB may be performed as appropriate on the premise of no damage to the accessory nerve. LCEI is safe and effective for lymph node dissection in level II. When the tumor is in the unilateral lobe, attention should be given to contralateral central lymph node dissection because of the high lymphatic metastasis rate.

## Data Availability

The datasets used or analyzed during the current study are available from the corresponding authors on reasonable request.

## Abbreviations

LCEI: Low-collar extended incision (LCEI)
PTC: Papillary thyroid carcinoma
BMI: Body mass index
